# Lateralization and cognitive systems

**DOI:** 10.3389/fpsyg.2014.01143

**Published:** 2014-10-08

**Authors:** Sebastian Ocklenburg, Marco Hirnstein, Christian Beste, Onur Güntürkün

**Affiliations:** ^1^Biopsychology, Institute of Cognitive Neuroscience, Ruhr-University BochumBochum, Germany; ^2^Bergen fMRI Group, Department of Biological and Medical Psychology, University of BergenBergen, Norway; ^3^Cognitive Neurophysiology, Department of Child and Adolescent Psychiatry, Faculty of Medicine of the Technische Universität DresdenDresden, Germany

**Keywords:** handedness, executive function, laterality, lateralization, hemispheric asymmetry, brain, language, brain structure

Lateralization of brain and behavior in both humans and non-human animals is a topic that has fascinated neuroscientists since its initial discovery in the mid of the nineteenth century (Broca, 1861; Dax, 1865; Oppenheimer, 1977; Ströckens et al., 2013). Hemispheric asymmetries are abundant in the anatomy, neurochemistry and cytoarchitecture of the vertebrate brain and over the decades, a number of cognitive abilities have been shown to heavily rely on lateralized processing in the brain, the most widely investigated being language (Corballis, 2012; Ocklenburg et al., 2013b). Other cognitive domains that depend on lateralized processing include emotional processing (Önal-Hartmann et al., 2012), face and body perception (Thoma et al., 2014), spatial attention (Duecker et al., 2013), fine motor skills (Arning et al., 2013) and memory (Habib et al., 2003)—just to name a few. However, the impact of lateralization of brain function is not limited to these “classical” domains of lateralization research. The efficiency of higher cognitive processes in the vertebrate brain does not only depend on the involved cognitive systems themselves, but also on earlier information processing stages (Knudsen, 2007). Therefore, functional hemispheric asymmetries in stimulus processing can affect the efficiency of virtually any cognitive domain. This principle has recently been demonstrated for executive functions mediated by fronto-striatal networks, including working memory processes (Beste et al., 2010a,b, 2011, 2012). Ocklenburg et al. (2011, 2012) could show that the efficiency of executive functions like response inhibition or task switching is modulated when functional hemispheric asymmetries affect stimulus processing.

Based on these observations, the present Frontiers in Cognition Research Topic aimed to further investigate the relationship of lateralization and cognitive systems in the vertebrate brain. Overall, the Research Topic encompasses more than 30 novel publications, ranging from Original Research Articles to Reviews and Mini Reviews, Perspective Articles and Hypothesis and Theory Articles. From the beginning, the present Research Topic was conceptualized with a comparative multi-disciplinary inter-species approach in mind. This idea is reflected in the broad diversity of animal models included in the Research Topic, ranging from invertebrates (Frasnelli, 2013) to different species of birds (Manns and Ströckens, 2014; Rugani et al., 2014) and primates (Hopkins et al., 2014). In addition to animal research, several studies examined how lateralization impacts the functioning of different cognitive systems in the human brain. For example, it was investigated how handedness is related to other brain functions such as language lateralization (Carey and Johnstone, 2014), approach/avoidance motivation (Hardie and Wright, 2014), perceptual asymmetries (Marzoli et al., 2014), semantic priming (Fagard et al., 2014), response speed in the orthogonal Simon task (Iani et al., 2014) and cognitive performance in general (Prichard et al., 2013; Scharoun and Bryden, 2014). These studies are complemented by a review article investigating how twin studies could be useful in the quest to understand the complex interrelations of lateralization and cognitive systems (Ooki, 2014) as well as by a large-scale anatomical work investigating the effect of handedness on the structure of the cerebral cortex (Guadalupe et al., 2014). The relation of structural and functional asymmetries was also the topic of review article that investigated the cortical microstructural basis of lateralized cognition (Chance, 2014). Moreover, several authors investigated auditory lateralization (e.g., Specht et al., 2014). For example, Hirnstein et al. (2014a; Erratum in Hirnstein et al., 2014b) investigated how language lateralization measured with the Dichotic Listening Task relates to cognitive performance. The same task was used in a new smartphone version by Bless et al. (2013) who investigated the feasibility of conducting research on the interaction between lateralization and cognitive systems using a smartphone application. With more than 5500 article views and an AM score of more than 50 by the time this editorial was written, this article has gained more online attention than almost any other work published in Frontiers in Cognition. Other authors investigated visual lateralization (Asanowicz et al., 2013; Pellicano et al., 2013; Helon and Króliczak, 2014), asymmetries in emotional processing (Propper and Brunyé, 2013; Grimshaw and Carmel, 2014), behavioral lateralization (Morton, 2013; Corbetta et al., 2014), and asymmetries in face (Coronel and Federmeier, 2014) and body representation (Hach and Schütz-Bosbach, 2014), as well as in word generation (Meyer et al., 2014) and word recognition (Izura et al., 2014). Finally, some authors also investigated the impact of lateralized processing on executive functioning, the topic which had initially inspired this Research Topic (Marsh et al., 2013; Ocklenburg et al., 2013a; Kéïta et al., 2014; Stock and Beste, 2014).

Taken together, the wide variety of cognitive systems in different species covered in the present Research Topic highlights the enormous importance of understanding how and why the vertebrate brain is asymmetrically organized for almost any subfield within cognitive neuroscience. We hope that the excellent papers assembled in the present Research Topic will help to stimulate more research aimed at understanding the complex mechanisms underlying the interaction between hemispheric asymmetries in stimulus perception and higher cognitive systems.

## Conflict of interest statement

The authors declare that the research was conducted in the absence of any commercial or financial relationships that could be construed as a potential conflict of interest.
